# AI in Cervical Cancer Cytology Diagnostics: A Narrative Review of Cutting-Edge Studies

**DOI:** 10.3390/bioengineering12070769

**Published:** 2025-07-16

**Authors:** Daniele Giansanti, Andrea Lastrucci, Antonia Pirrera, Sandra Villani, Elisabetta Carico, Enrico Giarnieri

**Affiliations:** 1 Centro TISP, Istituto Superiore di Sanità, Via Regina Elena 299, 00161 Rome, Italy; antonia.pirrera@iss.it; 2 Department of Allied Health Professions, Azienda Ospedaliero-Universitaria Careggi, 50134 Florence, Italy; andrea.lastrucci@unifi.it; 3 Department of Clinical and Molecular Medicine, Sant’Andrea University Hospital, Sapienza University of Rome, Via di Grottarossa 1035, 00189 Rome, Italy; svillani@ospedalesantandrea.it (S.V.); elisabetta.carico@uniroma1.it (E.C.)

**Keywords:** cytology, cytopathology, cancer, cervix, cervical, artificial intelligence

## Abstract

**Background:** The integration of artificial intelligence (AI) into cervical cancer diagnostics has shown promising advancements in recent years. AI technologies, particularly in the analysis of cytological images, offer potential improvements in diagnostic accuracy and screening efficiency. However, challenges regarding model generalizability, explainability, and operational integration into clinical workflows persist, impeding widespread adoption. **Aim:** This narrative review aims to critically evaluate the current state of AI in cervical cancer diagnostic cytology, identifying trends, key developments, and areas requiring further research. It also explores the potential for AI to improve diagnostic processes, alongside examining international guidelines and consensus on its adoption. **Methods:** A narrative review was conducted through a comprehensive search of PubMed and Scopus databases. Thirty studies published between 2020 and 2025 were selected based on their relevance. **Results:** The literature review reveals a growing interest in the application of AI for cervical cancer diagnostics, particularly in the automated interpretation. However, large-scale clinical adoption remains limited. Most studies are experimental or application-based in controlled settings. Consensus efforts and specific recommendations for this domain are still limited and not specific. Key barriers include limited model generalizability, lack of explainability, challenges in integration into clinical workflows, and regulatory and infrastructural constraints. **Conclusions:** A sustainable and meaningful integration of AI in cervical cancer diagnostics requires a unified framework that addresses both technical challenges and operational needs, supported by context-specific strategies and broader consensus-building efforts.

## 1. Introduction

### 1.1. Historical Foundations of Cervical Cancer Diagnostics

The history of cervical cancer diagnostics is deeply rooted in one of the most impactful public health advancements in oncology. At a time when invasive cervical cancer was among the leading causes of cancer death in women, the introduction of cytological screening profoundly changed the natural history of the disease [1]. In the 1920s, George Papanicolaou developed the Pap smear, a simple yet powerful test based on the microscopic examination of exfoliated cervical cells. Initially met with skepticism, the method gained wide acceptance in the 1950s and 1960s, when organized screening programs in North America and Europe demonstrated dramatic reductions in cervical cancer incidence and mortality [2]. For the first time, it became possible to routinely detect precancerous lesions—often asymptomatic—and treat them before progression to invasive disease. To complement cytological screening, colposcopy emerged as a key diagnostic tool [3]. First devised by Hans Hinselmann in 1925, colposcopy involves the magnified visual inspection of the cervix using a colposcope, allowing clinicians to identify abnormal epithelial patterns and guide targeted biopsies. The integration of colposcopy into diagnostic workflows during the latter half of the 20th century improved the precision of diagnosis following abnormal cytological results and became a standard component of the evaluation of cervical lesions.

The development of histopathological classifications, such as Cervical Intraepithelial Neoplasia (CIN) and, later, the Bethesda System for reporting cytological findings (introduced in the late 1980s), provided clinicians and laboratories with standardized terminologies [4]. These frameworks improved diagnostic consistency and facilitated clinical decision-making across different healthcare systems.

A decisive turning point came with the discovery and scientific validation of the human papillomavirus (HPV) as the primary etiological agent of cervical cancer. Through molecular and epidemiological research in the 1980s and 1990s, high-risk HPV types were conclusively linked to the development of cervical neoplasia [5]. This led to the introduction of HPV DNA testing as a diagnostic tool in the early 2000s, initially as a triage method for equivocal cytology and eventually as a primary screening test in several countries. HPV testing offered greater sensitivity than cytology in detecting high-grade lesions, particularly in women over 30 years of age, and helped stratify patient risk more effectively.

In parallel, the 1990s also saw the advent of the first computer-assisted cytology systems, such as PapNet and AutoPap [6,7,8], which represented early attempts to automate and standardize the interpretation of Pap smears. These systems used neural networks and image analysis algorithms to pre-screen slides, flagging potentially abnormal areas for human review. While their adoption remained limited due to cost, workflow integration issues, and skepticism in the clinical community, they paved the way for subsequent advances in digital cytology and the development of AI-enhanced diagnostics.

Over the span of a century, cervical cancer diagnostics evolved from purely morphological observation under a microscope to integrated strategies combining cytology, colposcopy, histopathology, and molecular virology. These innovations not only enabled earlier and more accurate diagnosis but also informed the development of clinical algorithms, screening guidelines, and follow-up protocols that remain central to modern practice.

### 1.2. The Impact of Digitalization and Artificial Intelligence on Cervical Cancer Diagnostics

Digitalization has significantly transformed cervical cancer diagnostics, marking a notable shift in both efficiency and diagnostic accuracy. Starting in the early 2000s, digital imaging has started to gradually replace manual microscopy, allowing for high-resolution visualization of cervical samples on screens rather than through traditional microscopes [9]. This enabled remote consultations, supported the rise of telepathology, and led to the development of digital archives, which improved the retrieval, sharing, and long-term storage of diagnostic data [10].

In parallel, the digitization of Pap smears allowed for the quantitative analysis of cellular features, enhancing objectivity and reproducibility in sample evaluation [11,12]. Tools for automated image analysis began assisting pathologists by flagging suspicious cells, which helped reduce variability between observers and standardize diagnoses. Still, human interpretation remained indispensable, especially in nuanced or borderline cases [13]. The more recent integration of Artificial Intelligence (AI) represents the next major evolution. Techniques such as Machine Learning (ML) and Deep Learning (DL) are being applied to the analysis of Pap smears, HPV testing, and colposcopy images, reflecting two distinct but complementary diagnostic approaches in cervical cancer detection.

The first approach is cytology-based, focusing on cellular-level evaluation through Pap smears, which remains central in detecting precancerous changes. The second approach is visual-based colposcopy, where gynecologists directly examine cervical tissue to identify visible lesions. These methods are supported by AI in different ways—automated cell classification in cytology, and image pattern recognition in colposcopy.

Although the HPV test is not cytology in the strict sense—since it involves molecular detection of high-risk viral DNA—it plays a fundamental role in screening strategies and is often combined with cytology for triage. AI is also beginning to assist in this context, for example, by analyzing viral patterns or integrating HPV status with image-based diagnostics to improve patient risk stratification.

Trained on large datasets, these AI models show promise and potential in identifying abnormal cells, offering decision support to clinicians and increasing pre-screening efficiency [14,15,16]. Nonetheless, AI is not without limitations. It requires diverse, high-quality training data to ensure generalizability and minimize bias, and often struggles with complex cases where clinical context is essential [17]. As such, AI should be seen as a complementary tool, enhancing but not replacing expert human evaluation.

Furthermore, the clinical integration of AI is still in progress. Many systems are undergoing validation and regulatory review, and widespread adoption depends on continued testing and refinement [18]. Despite these challenges, AI could offer practical advantages—from automating routine tasks to highlighting regions of interest and helping clinicians form a comprehensive picture of a patient’s risk profile. Recent approvals by regulatory bodies, such as the FDA, demonstrate the increasing adoption of AI for diagnostic tasks in cervical cancer screening [19,20].

### 1.3. Purpose

The diagnosis of cervical cancer involves critical challenges, including the subjective nature of cytological interpretation, the high workload in screening programs, and the complexity of integrating multiple diagnostic modalities such as HPV testing and colposcopy. These issues often lead to variability in accuracy, diagnostic delays, and resource constraints, especially in low-resource settings. Artificial Intelligence (AI) holds strong potential to overcome these barriers by enhancing diagnostic consistency, workflow efficiency and, ultimately, patient outcomes.

Cervical cytology remains the primary gateway to cervical cancer detection and triage in most screening programs worldwide, offering a standardized, scalable, and cost-effective approach—unlike colposcopy, which is more subjective, operator-dependent, and typically reserved for secondary assessment following abnormal screening results.

In light of recent advancements, it is important to map the current state of development and integration of Artificial Intelligence (AI) in cervical cancer diagnostic cytology. A narrative review of this evolution, with three key objectives, will help provide a comprehensive overview of the current progress in this field:

1. **Evolution of Scientific Output**: Examining recent publications on AI’s role in cervical cancer diagnostics, identifying major research milestones and advancements.
2. **Key Topics and Categorization**: Focusing on key themes in AI applications, such as automated cell analysis, image recognition, and diagnostic enhancement.
3. **Opportunities and Challenges**: Analyzing AI’s potential to optimize workflows, while also addressing ongoing challenges, like data biases, model accuracy, and regulatory barriers.

This review offers an up-to-date understanding of how AI is being integrated into cervical oncology workflows, highlighting practical benefits, challenges, and ongoing developments in the field. The focus is specifically on diagnostic cytology, as this remains a cornerstone of cervical cancer screening programs, and is where AI tools—particularly those involving image analysis and pattern recognition—have shown some of the most promising early results.

A narrative review is particularly suitable for this work, as it captures the recent, rapidly evolving landscape of AI integration in cervical cancer diagnostics. While systematic reviews may overlook newer trends and advancements due to their structured nature, a narrative review allows for a more fluid and comprehensive exploration, acknowledging the complexity and dynamic nature of the field.

Focusing on the last five years is especially relevant, as this period has seen a surge in AI applications due to improved computational power, the availability of large annotated datasets, and growing regulatory interest in digital pathology tools. These developments have significantly accelerated translation from research to practice, making a timely synthesis both necessary and impactful.

## 2. Methods

This narrative review was conducted by searching the PubMed and Scopus databases for recent articles exploring the application of artificial intelligence (AI) in cervical cancer. The ANDJ checklist for the narrative review was used. The search was designed to include studies from various perspectives, such as early detection, risk stratification, and treatment response. PubMed was chosen as the primary database due to its focus on biomedical literature, while Scopus was also consulted to ensure a broader representation, including interdisciplinary and international research.

The following search terms were used:

**Search string**

*((Cervix cancer[Title/Abstract]) OR (Cervix carcinoma[Title/Abstract]) OR (Cervix dysplasia[Title/Abstract]) OR (Cervical intraepithelial neoplasia [Title/Abstract]) OR (Cervical cancer pathophysiology[Title/Abstract]) OR (Cervix HPV infection[Title/Abstract]) OR (Cervix biopsy and cancer diagnosis[Title/Abstract]) OR (Cervix cancer staging[Title/Abstract]) OR (Cervix cancer early detection[Title/Abstract]) OR (Cervix cancer metastasis[Title/Abstract]) OR (Cervical cancer[Title/Abstract]) OR (Cervical carcinoma[Title/Abstract]) OR (Human papillomavirus (HPV) and cervical cancer[Title/Abstract]) OR (Cervical cancer screening[Title/Abstract]) OR (Cervical dysplasia[Title/Abstract]) OR (Cervical cancer prevention[Title/Abstract]) OR (HPV vaccination and cervical cancer[Title/Abstract]) OR (Cervical cancer treatment[Title/Abstract]) OR (Risk factors for cervical cancer[Title/Abstract]) OR (Cervical cancer stages[Title/Abstract]) OR (Cervical cancer survival rates[Title/Abstract]) OR (Cervical cancer prognosis[Title/Abstract]) OR (Cervical cancer immunotherapy[Title/Abstract]) OR (Cervical cancer biomarkers[Title/Abstract]) OR (Early detection of cervical cancer[Title/Abstract]))AND ((Machine learning[Title/Abstract] OR Deep learning[Title/Abstract]) OR (Neural networks[Title/Abstract] OR Predictive modeling[Title/Abstract]) OR (Natural language[Title/Abstract] OR Data science[Title/Abstract]) OR (AI applications[Title/Abstract] OR Reinforcement learning[Title/Abstract]) OR (Supervised learning[Title/Abstract] OR AI models[Title/Abstract])) AND ((Cytology[Title/Abstract]) OR (Cytopathology[Title/Abstract]))*

Although the search string contains several closely related or similar terms, there are no exact duplicates among the keywords. Terms like *“Cervix cancer”* and *“Cervical cancer”*, or *“Cervix dysplasia”* and *“Cervical dysplasia”*, may appear conceptually overlapping, but they are technically distinct and are intentionally included to capture variations in terminology used across the literature.

This is a common and necessary practice in comprehensive database searches, especially in biomedical fields, where slight changes in phrasing can affect search results. For example, some articles may use *“cervical”* (adjective), while others refer to *“cervix”* (noun) explicitly. Including both ensures that the search retrieves the widest possible range of relevant publications.

In conclusion, while there are no literal repetitions, the presence of semantically similar terms is deliberate and improves the sensitivity of the search strategy.

The articles considered were limited to those published in the last five years to ensure relevance to current trends and technological advancements in AI applications for cervical cancer. Notably, the COVID-19 pandemic played a pivotal role in accelerating the development and application of artificial intelligence in healthcare. The urgency of adapting to new care delivery models, such as telemedicine and remote diagnostics, prompted a significant push towards AI-based solutions. During this period, AI technologies demonstrated their capacity to address critical healthcare challenges, including efficient screening and diagnosis of cervical cancer, where remote solutions and AI-powered systems became increasingly important. For this reason, the accelerated adoption of AI during the pandemic highlighted its potential to innovate and improve patient care, particularly in settings where physical access to healthcare professionals was limited. Only studies that applied AI technologies to clinical settings or patient care were included. Studies primarily focused on theoretical or computational aspects of AI without practical application were excluded, as the aim of this review was to highlight studies with clear, real-world relevance, such as AI-based diagnostic tools, predictive modeling, and decision-support systems.

The review prioritized studies that addressed key issues in cervical cancer, such as early detection, diagnostic accuracy, treatment optimization, and patient outcomes. Papers that primarily discussed AI algorithms in a generic or theoretical context, without direct links to cervical cancer management, were excluded.

The selection of articles was driven by their contribution to advancing AI in the clinical and practical management of cervical cancer. Studies that demonstrated AI’s integration into healthcare workflows, its potential to solve existing challenges in cervical cancer care, and its applicability to improving patient outcomes were emphasized. This narrative review provides a comprehensive overview of the most recent and relevant research, rather than focusing on a rigid synthesis of study quality, as would be the case in a systematic review. While systematic reviews are valuable for summarizing established knowledge, they can be too rigid and limiting in a field characterized by rapid innovation and emerging technologies. The flexibility of a narrative approach allows for the inclusion of cutting-edge studies that highlight the evolving role of AI in the management of cervical cancer, capturing the nuances and advancements in this fast-moving area of research.

## 3. Results

The sections are organized coherently along with the objectives and results. Therefore, Section 1 provides a bibliometric analysis conducted on PubMed. Section 3.2 focuses on emerging themes, while Section 3.3 presents emerging opportunities and challenges.

### 3.1. Bibliometric Trends

A bibliometric analysis conducted on PubMed reveals some interesting trends when comparing the integration of AI in cervical cancer diagnostics with the broader field of AI in cytology and cytopathology. Box 1 reports the two search keys employed to retrieve relevant literature for this study. Key 1 specifically targets publications at the intersection of artificial intelligence (AI) and cervical cancer cytology, focusing on research that applies AI techniques directly to cervical cancer diagnosis through cytological methods. Key 2 encompasses a broader scope, covering AI applications across the entire field of cytology, regardless of cancer type, thereby capturing a wide range of studies involving AI in cytological analysis. These carefully constructed search keys ensured comprehensive retrieval of both focused and general AI-related cytology research, enabling a robust comparative analysis.

**Box** **1** **Used search keys.**

*((Cervix cancer[Title/Abstract]) OR (Cervix carcinoma[Title/Abstract]) OR (Cervix dysplasia[Title/Abstract]) OR (Cervical intraepithelial neoplasia [Title/Abstract]) OR (Cervical cancer pathophysiology[Title/Abstract]) OR (Cervix HPV infection[Title/Abstract]) OR (Cervix biopsy and cancer diagnosis[Title/Abstract]) OR (Cervix cancer staging[Title/Abstract]) OR (Cervix cancer early detection[Title/Abstract]) OR (Cervix cancer metastasis[Title/Abstract]) OR (Cervical cancer[Title/Abstract]) OR (Cervical carcinoma[Title/Abstract]) OR (Human papillomavirus (HPV) and cervical cancer[Title/Abstract]) OR (Cervical cancer screening[Title/Abstract]) OR (Cervical dysplasia[Title/Abstract]) OR (Cervical cancer prevention[Title/Abstract]) OR (HPV vaccination and cervical cancer[Title/Abstract]) OR (Cervical cancer treatment[Title/Abstract]) OR (Risk factors for cervical cancer[Title/Abstract]) OR (Cervical cancer stages[Title/Abstract]) OR (Cervical cancer survival rates[Title/Abstract]) OR (Cervical cancer prognosis[Title/Abstract]) OR (Cervical cancer immunotherapy[Title/Abstract]) OR (Cervical cancer biomarkers[Title/Abstract]) OR (Early detection of cervical cancer[Title/Abstract]))AND ((Machine learning[Title/Abstract] OR Deep learning[Title/Abstract]) OR (Neural networks[Title/Abstract] OR Predictive modeling[Title/Abstract]) OR (Natural language[Title/Abstract] OR Data science[Title/Abstract]) OR (AI applications[Title/Abstract] OR Reinforcement learning[Title/Abstract]) OR (Supervised learning[Title/Abstract] OR AI models[Title/Abstract])) AND ((Cytology[Title/Abstract]) OR (Cytopathology[Title/Abstract]))* ^1^*((Machine learning[Title/Abstract] OR Deep learning[Title/Abstract] OR Neural networks[Title/Abstract] OR Predictive modeling[Title/Abstract] OR Natural language[Title/Abstract] OR Data science[Title/Abstract] OR AI applications[Title/Abstract] OR Reinforcement learning[Title/Abstract] OR Supervised learning[Title/Abstract] OR AI models[Title/Abstract]))* *AND* *((Cytology[Title/Abstract]) OR (Cytopathology[Title/Abstract]))*^1^ Note: While several terms in the search string may appear semantically similar (e.g., “*Cervix cancer*” vs. “*Cervical cancer*”, or “*Cervix dysplasia*” vs. “Cervical dysplasia”), they are not redundant. Rather, their inclusion reflects intentional variation to accommodate differences in how concepts are labeled across scientific literature. The use of both noun-based and adjective-based terms (e.g., “*cervix*” vs. “*cervical*”) broadens the scope of the search and enhances its sensitivity, ensuring that relevant articles are not missed due to terminological differences.

**Figure 1:**
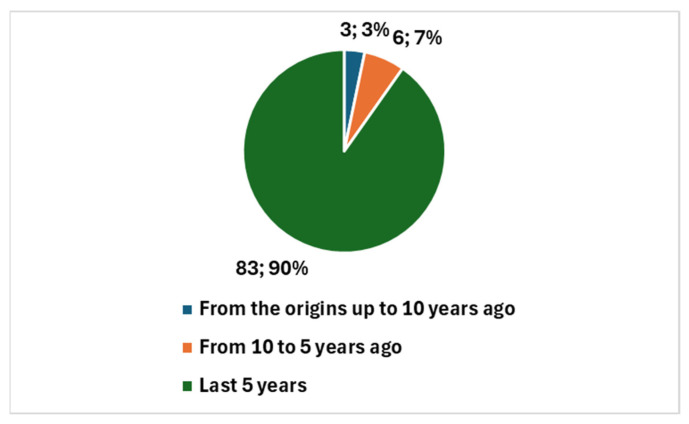
Historical trends in scientific publications on cervical cancer and AI in cytology, with a focused analysis of developments over the past five and ten years.

**Figure 2:**
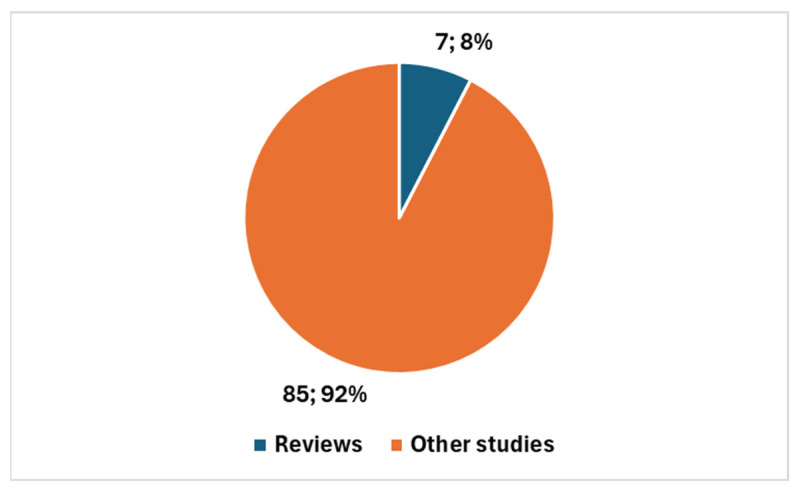
Distribution of Review and Original Research Studies on Cervical Cancer and AI in Cytology.

Specifically, in the case of cervical cancer, 92 studies have been published since 2011, with a notable surge in recent years. Of these, 89 studies were published in the last decade, representing 96.74% of the total; furthermore, 83 studies have been published in the past 5 years, accounting for 90.22% of the total (Figure 1). This rapid increase points to a growing interest and investment in AI for cervical cancer, likely fueled by technological advancements and the pandemic’s impact on research priorities. However, only seven studies (or 7.61%) are reviews, suggesting that the field is still developing, with a focus on original research rather than comprehensive overviews (Figure 2).

**Figure 3:**
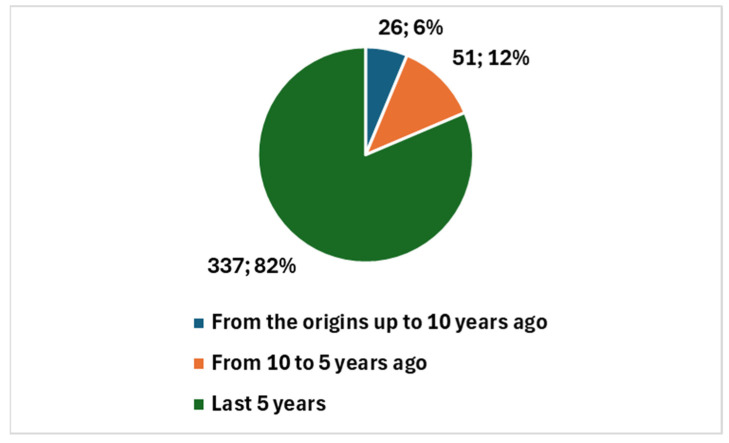
Temporal Trends in Scientific Publications on Cytology and AI: Focus on the Past Five and Ten Years.

**Figure 4:**
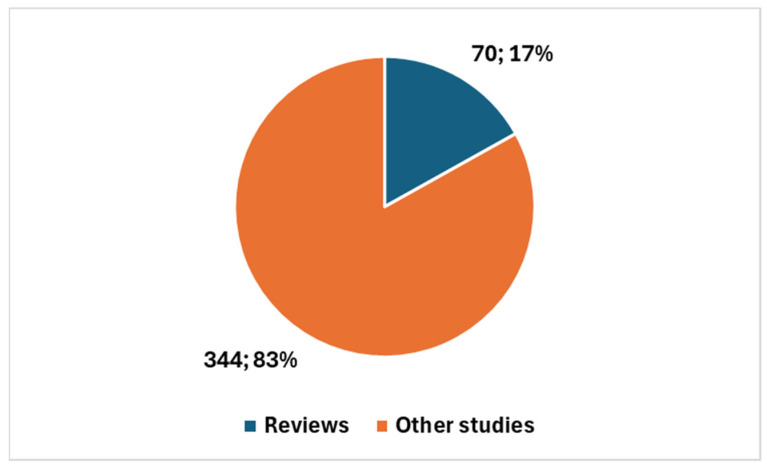
Proportion of Review and Original Studies in Cytology Related to Cervical Cancer and AI.

In comparison, the broader scientific field of AI in cytology and cytopathology (see key 2 in Box 1) includes 414 studies since 1983, showing a steady growth trajectory. Narrowing the focus to the last 10 years, 388 studies (or 93.72%) have been published and, over the last 5 years, 337 studies (or 81.40%) were published (Figure 3). This indicates a longer-established interest in AI within this field, with a larger body of research accumulated over time. Additionally, review articles make up 16.91% of the studies in the broader field (70 review articles) (Figure 4), reflecting the more mature state of this area, where comprehensive reviews are necessary to synthesize and summarize existing research.

When considering the proportion of cervical cancer studies in the broader context of AI in cytology and cytopathology, the 92 studies on cervical cancer represent 22.2% of the total 414 studies in this area. These 92 studies represent 22.2% of the total 414 studies. Given the vast number of cancer types and other focuses, different from cancer, within cytology, this 22% is a noteworthy figure, highlighting the growing attention dedicated to cervical cancer amidst a wide range of other cancer types, such as thyroid, urinary bladder, lung, breast, pleural effusion, ovary, pancreas, and prostate.

A notable aspect emerging from the trend analysis of AI applications in cytology is the disproportionately high focus on cervical cancer, which accounts for approximately 22% of the research output in this field despite the wide variety of cancers and other conditions studied through cytological methods. This prominence is not unexpected considering that cervical cytology represents a substantial portion of the global cytopathology laboratory workload, with a significant share dedicated to cervical cancer screening—primarily through conventional Papanicolaou (Pap) smears and Liquid-Based Cytology (LBC)—optimizing diagnostic efficiency in this area remains a critical priority.

This significant workload has long motivated the pursuit of automation in cervical cytology diagnostics. In many healthcare settings, initial screening of cytological slides is performed by specialized personnel who are often not medical doctors, commonly referred to as screeners. These screeners review large volumes of slides to identify suspicious or abnormal findings, which are then referred to expert cytopathologists for confirmation. However, the availability of trained screeners can be limited, particularly in low-resource settings, due to the challenges involved in their recruitment and training.

Therefore, the integration of Artificial Intelligence (AI) into cervical cytology represents a strategic approach aimed at addressing both the high volume of screening demands and the shortage of specialized personnel. AI tools have the potential to assist or partially automate the identification of abnormal cells, thereby enhancing efficiency, reducing human error, and potentially expanding access to quality cervical cancer screening in underserved areas.

The high percentage of AI research focused on cervical cytology reflects this operational necessity, as well as the clear clinical importance of early cervical cancer detection. This context underlines the rationale behind the concentration of AI applications in cervical cytology and highlights the value of continued innovation and validation efforts in this domain.

Overall, this comparison highlights that, while the field of AI in cervical cancer diagnostics is rapidly expanding—particularly in the last 5 years, with 90.22% of studies published compared to 81.40% in the broader field—the broader field of AI in cytology and cytopathology has a longer history and more established foundation. The sharp rise in cervical cancer research signals a promising, emerging area of focus, while the broader field’s longer history underscores its more mature and diversified nature.

### 3.2. Themes and Categorization

The literature review based on 30 studies [21,22,23,24,25,26,27,28,29,30,31,32,33,34,35,36,37,38,39,40,41,42,43,44,45,46,47,48,49,50] shows that cervical cancer screening increasingly relies on artificial intelligence (AI) and deep learning techniques to enhance early detection accuracy and improve patient outcomes.

Multiple studies [21,22] have developed and validated convolutional neural networks (CNNs), Swin Transformers, and ensemble deep learning models to analyze cervical cytology images, achieving higher precision in classifying precancerous and cancerous lesions compared to traditional manual screening.

Convolutional neural networks (CNNs) are specialized deep learning models particularly effective for image analysis because they automatically learn to identify important visual features such as shapes and textures in medical images, enabling precise detection of abnormalities. Swin Transformers, a newer AI architecture, apply an attention mechanism that allows the model to consider relationships between different parts of the image, capturing more complex patterns across the entire visual field. Ensemble deep learning models combine the outputs of multiple AI models to improve accuracy and robustness by leveraging the strengths of each individual model.

The identification of novel molecular biomarkers such as the hypermethylation marker ZSCAN18 [24] combined with machine learning-based diagnostic models further improves early detection and risk stratification.

Machine learning models learn from data to identify patterns linked to disease risk, enabling more accurate prediction and personalized patient management.

For instance, the SMART-HPV model [25] uses full-genotyping of high-risk HPV strains with machine learning to better stratify patients’ risk and guide clinical management, while additional cohort-based studies [39] validate these approaches for populations with ambiguous cytology results.

The availability of large, well-annotated cervical cytology image datasets [27,30,43] supports the development and training of robust AI models, while innovative methods, like contrastive self-supervised learning [28] and self-supervised learning-based cytology classification [49], enable effective model training even with limited labeled data, a crucial advantage for resource-limited settings.

Self-supervised learning techniques allow AI models to learn useful representations from unlabeled data by contrasting similar and dissimilar examples. This reduces dependency on costly expert annotations, making it feasible to develop effective AI in contexts where labeled datasets are scarce.

Addressing challenges such as confusing labels and morphological heterogeneity in cervical pathology images through correction techniques [26,29,40] improves classification reliability and reduces false positives or negatives.

Liquid-based cytology combined with AI-driven image analysis [21,36,44] has demonstrated improved sensitivity and specificity compared to traditional Pap smear interpretation, offering scalable solutions for mass screening.

Explainable AI (xAI) methods [46] and semi-supervised learning approaches [47] enhance model transparency and interpretability, enabling clinicians to better understand and trust AI-based decisions.

Explainable AI helps to clarify how AI models reach their conclusions, which is essential in clinical settings to ensure decisions are transparent and clinically valid. Semi-supervised learning combines a small amount of labeled data with a larger amount of unlabeled data, improving model training efficiency and accuracy.

Furthermore, the integration of transfer learning and attention mechanisms [37], along with advanced segmentation techniques [45,50], refines the detection and classification of cervical abnormalities.

Transfer learning leverages knowledge gained from previously trained models on related tasks to improve performance on new but similar tasks, reducing the need for large datasets. Attention mechanisms help models focus on the most relevant parts of an image, enhancing diagnostic precision.

Emerging research also explores the use of cervical liquid-based cytology samples as a source for microbiome profiling [31], opening avenues for dual diagnosis and a more comprehensive understanding of cervical health.

Reinforcement learning algorithms [32] and automated multiplex PCR genotyping platforms augmented by AI and data science [41] contribute to personalized, precise cervical cancer diagnostics.

Collectively, these technological advances position AI as a transformative tool in cervical cancer screening by enabling high-accuracy, risk-stratified diagnosis and facilitating implementation in diverse clinical contexts, including low-resource environments, ultimately promising improved patient outcomes and more efficient healthcare workflows [33,34,38]. The inclusion of references [23,25,34], all identified through the proposed search composite key, is motivated by their clear demonstration of AI applications that complement and extend beyond cytology. These studies internally discuss and address the potential and complementary roles of AI applied to other diagnostic tools and methods beyond cytology in enhancing screening approaches, highlighting their added value and integration potential. Reference [23] applies a hybrid deep learning model to colposcopy images, adding a visual diagnostic layer beyond cytological smears. This image-based AI approach uses Swin Transformer architectures to analyze cervical morphology in situ, enhancing detection of lesions that cytology alone might miss. Dong et al. [25] employ machine learning within the SMART-HPV framework to analyze HPV genotyping data, enabling refined molecular risk stratification that complements the cellular focus of cytology. Goldstein et al. [34] provide a comprehensive overview of AI’s transformative role, emphasizing that AI-driven technologies extend beyond cytology to reduce false negatives, lower costs, and improve global accessibility. Together, these references highlight the multidimensional expansion of cervical cancer screening through AI—from image analysis to molecular profiling and systemic innovations.—justifying their inclusion as essential contributions to this evolving field.

Table 1 below synthesizes these contributions [21,22,23,24,25,26,27,28,29,30,31,32,33,34,35,36,37,38,39,40,41,42,43,44,45,46,47,48,49,50], summarizing each study’s methodological focus, healthcare application, and relevance for the integration of AI in cervical cancer diagnostics. The last column of Table 1 reports detailed information regarding the application of artificial intelligence in each study. This includes the specific AI methodologies employed—ranging from convolutional neural networks, transfer learning, semi-supervised and self-supervised learning, to explainable AI frameworks—and their targeted functions, such as cell image representation, lesion classification, risk stratification, segmentation, and automated HPV genotyping. These descriptions elucidate how AI technologies are integrated within cervical cancer screening workflows and highlight their innovative contributions to improving diagnostic accuracy and efficiency.

### 3.3. Emerging Opportunities and Challenges

#### 3.3.1. Opportunities in AI Integration for Cervical Cancer Diagnostics

The application of artificial intelligence (AI) in cervical cancer diagnostics presents multiple opportunities to enhance screening and triage processes. One of the most significant advantages is the automation and standardization of cytological interpretation. AI-based models using convolutional neural networks and vision transformers have demonstrated robust performance in classifying cervical images and Pap smears, reducing interobserver variability and increasing diagnostic consistency [21,22,23,24,25,26,27,28,29,47,48,49]. AI also offers the possibility of enhanced triage and risk stratification by integrating imaging data with clinical, cytological, virological, and molecular information [30,31,32,33,34,35,36,37]. This multimodal approach supports more accurate patient categorization and personalized management, potentially improving early detection while reducing overtreatment. In addition, several studies have shown the potential for AI to expand access to diagnostics in low-resource settings. Algorithms embedded in mobile devices or cloud platforms can assist less experienced clinicians by providing real-time decision support during diagnostics [41,42,43]. Such technologies may bridge gaps in geographic and workforce disparities, particularly in areas with limited access to expert care. AI tools are also being designed to work within digital workflows, enhancing operational efficiency by automating data extraction, feature analysis, and quality control in large-scale screening programs [25,26,44,45]. When properly validated and integrated, these tools can support timely and scalable deployment in both primary care and specialized settings.

#### 3.3.2. Challenges in AI Integration for Cervical Cancer Diagnostics

Despite promising developments, the clinical translation of AI faces several important challenges. One primary concern is generalizability. Many algorithms are trained on limited or homogeneous datasets, often from single institutions, raising concerns about their applicability across diverse populations and imaging systems [50]. This limitation may lead to biased predictions or reduced performance in real-world settings if not rigorously addressed through external validation.

Another critical issue is model explainability and transparency. Many AI models function as “black boxes,” making it difficult for clinicians to understand or trust the rationale behind their outputs [38,39,40]. The lack of interpretability hinders clinical adoption and raises concerns regarding safety and accountability.

Regulatory and legal challenges also persist. Most AI tools used in cervical cancer diagnostics are still in early development phases, and few have received regulatory approval. Issues such as data privacy, liability in case of misdiagnosis, and the absence of clear guidelines for clinical use remain unresolved [36,39,50].

Furthermore, the integration of AI into clinical practice requires cultural and infrastructural change. This includes training healthcare providers, adapting workflows, and fostering collaboration between clinicians, data scientists, and public health authorities [27,28,42,46]. Without this coordinated effort, even high-performing models may not yield measurable benefits in patient outcomes.

Lastly, socio-technical barriers—including disparities in digital infrastructure, resistance to automation, and lack of funding—can slow down implementation, particularly in under-resourced settings [43,45].

## 4. Discussion

The discussion is organized into three sections. Section 4.1 presents the added value of the narrative review and its highlights. Section 4.2 discusses the integration of AI into cervical cancer diagnostics and cytology, facing the market growth and addressing the challenges faced and highlighting areas for further development, based on comparisons with the existing literature. Section 4.3 reports a sample of commercial experiences. Finally, Section 4.4 presents the limitations.

### 4.1. Added Values and Highlights

This review aimed to address the critical challenges faced in the diagnosis of cervical cancer by providing a comprehensive overview of the current state of Artificial Intelligence (AI) applications in this field. The subjective nature of cytological interpretation, the high volume of samples requiring screening, the complexity of ambiguous cases, and the integration of multiple diagnostic modalities have long represented significant barriers to accurate and timely diagnosis. Through a systematic examination of recent advancements, this review highlights how AI-driven tools can potentially bridge these gaps by improving diagnostic accuracy, reducing variability, and alleviating workload burdens in cytopathology laboratories.

By synthesizing evidence on AI models designed to assist or automate aspects of cervical cytology, this work underscores the promise of these technologies to augment human expertise, facilitate earlier detection, and streamline clinical workflows. Nonetheless, it also recognizes ongoing challenges, such as the need for standardization, data quality assurance, and thoughtful clinical integration, to ensure that AI tools deliver safe and effective support in real-world settings.

#### 4.1.1. Added Value

**Figure 5:**
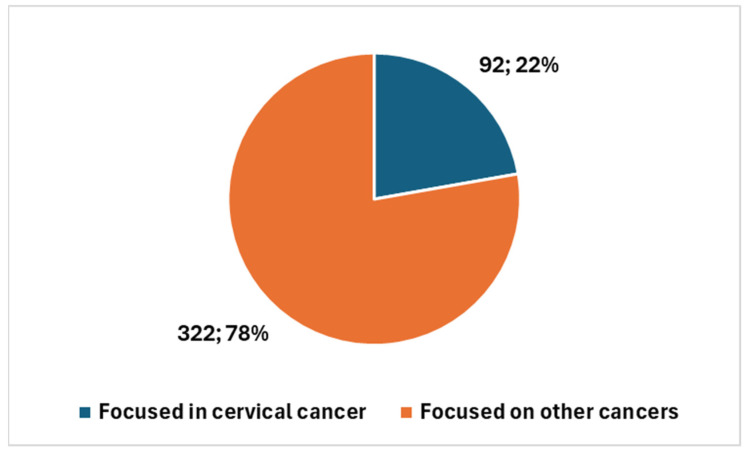
Breakdown of Studies on Cytology and AI Versus Cervical Cancer and AI.

Artificial intelligence (AI) is undergoing rapid evolution in the field of cytological diagnostics for cervical cancer. While this area remains a specialized field within the broader field of cytology, the growth in the past 5 to 10 years has been noteworthy, signaling significant advances in the integration of AI tools. The 22% (Figure 5) of studies focused on cervical cancer and AI is a significant proportion, especially when considering the wide range of other non-gynecological cancer types analyzed in cytology with AI, such as thyroid, urinary bladder, lung, breast, pleural effusion, ovary, pancreas, and prostate [51], and other possible focuses different from cancer. This highlights the importance of cervical cancer research within the broader context of AI in cytology and cytopathology.

As is often the case in fast-evolving scientific fields that have not yet reached full stabilization, it is essential to recognize the emerging themes, as well as the opportunities and challenges that will define the future of this area. This is precisely why we have chosen to develop a narrative review rather than a systematic review. A narrative review is better suited to capturing the nuances of a rapidly changing field, as it provides a more flexible framework for discussing broad trends and ongoing developments. Systematic reviews, on the other hand, are typically designed to address specific, well-defined research questions, which would assume that the field has already reached a certain level of maturity and stabilization.

This choice is further supported by the fact that, despite the growing interest and investment in AI for cervical cancer diagnostics, only one recent systematic review focused on the very specific topic of accuracy and analytical validity was found in PubMed [33].

Interestingly, this review addressed AI not just in cervical cancer but across a range of cancer types. This highlights the early-stage nature of AI in cervical cancer diagnostics, where the focus is still predominantly on original research, algorithm development, and validating AI models for specific applications rather than on comprehensive, well-established overviews.

One of the key added values of this narrative review is that it identifies the hot topics of interest in the cutting-edge research surrounding AI in cervical cancer diagnostics. These are the areas that are generating the most excitement and potential for transformative change in the field. By highlighting these focal points, we aim to provide a roadmap for future research that will help guide the direction of AI innovations in cervical cancer diagnostics.

Furthermore, this review also addresses the opportunities that are starting to emerge in this area. AI has the potential to revolutionize cervical cancer diagnostics by improving accuracy, consistency, and efficiency in screenings and diagnoses. AI-driven tools can enhance the interpretation of cytological images, reduce interobserver variability, and enable more personalized and precise treatment strategies. For example, AI can integrate various data sources, such as clinical, cytological, virological, and molecular information, to improve patient risk stratification and enable early detection, ultimately reducing unnecessary treatments.

A second added value is that this review also identifies the emerging opportunities in the field. As AI continues to evolve, it can significantly improve the personalization of treatment strategies by integrating various forms of patient data, which ultimately leads to more precise and targeted healthcare delivery. This ability to integrate diverse data points offers a more comprehensive view of a patient’s condition, improving clinical decision-making and enhancing patient outcomes.

A third added value is that this review highlights the challenges and open problems that need to be addressed. These hurdles are critical for the full integration of AI into clinical practice. For instance, issues such as the generalizability of AI models, data privacy concerns, model transparency, and the lack of clear regulatory guidelines pose significant barriers. These challenges must be overcome for AI tools to achieve widespread adoption and for their benefits to be realized across diverse healthcare settings. Identifying and understanding these challenges is essential for researchers and healthcare providers as they work toward the practical implementation of AI in cervical cancer diagnostics.

Overall, the opportunities presented by AI are vast, but so too are the challenges. It is these very challenges and unresolved issues that provide the foundation for a broader discussion in this field. Addressing them will require coordinated efforts from researchers, clinicians, data scientists, and public health authorities. Only through this collaborative approach can we expect to see meaningful progress in the integration of AI into clinical workflows, improving patient outcomes and revolutionizing the way cervical cancer is diagnosed and treated. Through this narrative review, we hope to contribute to this ongoing dialogue, offering insights into both the potential and the complexities of AI in this critical area of healthcare.

#### 4.1.2. Highlights

The integration of artificial intelligence (AI) into cervical cancer diagnostics is rapidly gaining momentum, with recent studies showcasing significant advancements. A bibliometric analysis of PubMed-indexed studies highlights a surge in research, especially in the last five years, indicating increasing scholarly interest in the field. While 92 studies specifically on AI in cervical cancer have been published since 2011, 90% of them have emerged within the last five years. This reflects a relatively new but rapidly growing focus, contrasting with the broader field of cytology and cytopathology, which has a longer history and a greater proportion of review articles. However, only seven studies (or 7.61%) are reviews, suggesting that the field is still developing, with a focus on original research rather than comprehensive overviews. This trend underscores the early-stage nature of AI applications in cervical cancer diagnostics.

Opportunities for AI integration are abundant. One of the most promising aspects is the automation and standardization of cytological interpretation. AI-based models using convolutional neural networks (CNNs) and vision transformers have demonstrated strong performance in classifying cervical images and Pap smears, offering a significant reduction in interobserver variability and enhancing diagnostic consistency [21,22,23,24,25,26,27,28,29,47,48,49]. Furthermore, AI facilitates enhanced triage and risk stratification by integrating various forms of data, such as clinical, cytological, virological, and molecular information [30,31,32,33,34,35,36,37]. This multimodal approach supports more precise patient categorization and personalized management, improving early detection while minimizing unnecessary treatments. In low-resource settings, AI has the potential to expand access to diagnostics by embedding algorithms in mobile devices or cloud platforms, providing real-time decision support to clinicians with varying levels of expertise [41,42,43]. These tools can help bridge geographic and workforce disparities, particularly in areas with limited access to expert care. Additionally, AI tools can streamline operational processes by automating data extraction, feature analysis, and quality control within large-scale screening programs. When properly validated and integrated into digital workflows, such tools can enhance efficiency and enable scalable deployment in both primary care and specialized healthcare settings [25,26,44,45].

Despite these promising advancements, several challenges hinder the seamless integration of AI into clinical practice. One key issue is generalizability. Many AI algorithms are trained on narrow, homogenous datasets, often from single institutions, which raises concerns about their applicability to diverse populations and imaging systems [50]. This limitation could result in biased predictions or reduced performance in real-world clinical environments if not addressed through extensive external validation. Another significant hurdle is the lack of model explainability and transparency. Many AI systems function as “black boxes,” which complicates clinicians’ ability to trust or understand the rationale behind the models’ outputs [38,39,40]. The absence of interpretability remains a barrier to widespread adoption, as it poses risks to patient safety and accountability in medical decision-making. Regulatory and legal challenges also need to be addressed. Currently, most AI tools for cervical cancer diagnostics are still in early stages, and only a few have received regulatory approval. Issues such as data privacy, liability in case of misdiagnosis, and the absence of standardized guidelines for clinical use must be resolved before these tools can be widely implemented [36,39,50]. Furthermore, the integration of AI into clinical practice requires a cultural and infrastructural shift. Healthcare providers must be trained, workflows adapted, and collaboration between clinicians, data scientists, and public health authorities fostered to ensure smooth AI adoption [27,28,42,46]. Without these coordinated efforts, even the most high-performing AI models may not translate into meaningful patient outcomes. Lastly, socio-technical barriers—including disparities in digital infrastructure, resistance to automation, and insufficient funding—can slow AI implementation, particularly in under-resourced settings [43,45]. Addressing these barriers will be critical to the successful integration of AI into global cervical cancer diagnostics.

### 4.2. Overcoming Challenges and Accelerating Growth: Integrating AI into Cervical Cancer Diagnostics and Cytology

The clinical translation of AI in cervical cancer diagnostics faces several important challenges that must be addressed for successful integration:

- **Generalizability:** Many algorithms are trained on narrow, homogeneous datasets, limiting their applicability to diverse populations and imaging systems, which can lead to biased predictions or poor real-world performance if not validated externally.
- **Model Explainability and Transparency:** AI models often act as “black boxes,” making it hard for clinicians to understand or trust the rationale behind the outputs, which hinders adoption and raises safety concerns.
- **Regulatory and Legal Challenges:** Most AI tools in cervical cancer diagnostics are in early stages, with few receiving regulatory approval. Issues like data privacy, liability in case of misdiagnosis, and a lack of clear clinical guidelines remain unresolved.
- **Cultural and Infrastructural Integration:** Implementing AI requires substantial changes in clinical workflows, including training healthcare providers and fostering collaboration between clinicians, data scientists, and public health authorities. Without these efforts, AI tools may not improve patient outcomes.
- **Socio-Technical Barriers:** Digital infrastructure disparities, resistance to automation, and insufficient funding, particularly in under-resourced areas, can slow down the implementation of AI.

These challenges highlight the importance of coordinated efforts to ensure that AI can be effectively integrated into clinical practice.

It is important to align the *market growth and the expansion of applications in this sector* with practical solutions to these challenges.

#### 4.2.1. Market Growth and Expanding Applications of AI in Cervical Cytology

The integration of artificial intelligence (AI) into cervical cancer screening is witnessing significant momentum, with the market expected to grow substantially over the coming decade. According to Future Market Insights, the cervical cancer screening market in the UK alone is projected to expand at a compound annual growth rate (CAGR) of 13.4% from 2025 to 2035, driven largely by the adoption of digital health technologies and AI tools in diagnostic workflows [52].

This economic trajectory mirrors the broader surge in scientific activity and clinical application of AI in cervical cytology. A recent bibliometric analysis in concordance with a part of our analysis highlighted a notable increase in publications on AI applications in cervical cancer diagnostics, confirming that this is not only an emergent field but one gaining consistent academic and clinical traction [53].

In parallel, large-scale studies have demonstrated the clinical effectiveness and scalability of AI tools. For instance, in a population-based study involving over 700,000 women in China, an AI-assisted cytology system achieved a concordance rate of 94.7% with human cytologists, alongside a 5.8% increase in sensitivity compared to manual screening [54]. These findings underscore the potential of AI to enhance diagnostic accuracy, particularly in high-throughput or resource-constrained environments.

High-performance metrics are also evident in more controlled studies. A deep learning model recently validated on a dataset of 16,000 participants reported an area under the curve (AUC) of 0.947, with a sensitivity of 94.6% and specificity of 89.0%, underscoring the diagnostic robustness of these tools [44].

The availability of high-quality public datasets further contributes to the expansion of AI in cervical cytology. For example, a new dataset recently supports the development and benchmarking of AI models by offering large, well-annotated cytology images for research and clinical testing [27].

Taken together, these trends suggest that the convergence of market forces, scientific advancements, and clinical validation is accelerating the integration of AI into cervical cancer diagnostics. The growing availability of data, coupled with increasing regulatory interest and the need for scalable solutions in both high- and low-resource settings, positions this field at a pivotal point of transformation.

#### 4.2.2. Towards Sustainable AI Integration: Addressing Technical, Operational, and Socio-Technical Challenges for Broader and Targeted Applications in Healthcare

When considering the integration of AI into digital cytology and cytopathology [55,56] more broadly—not just in the context of cervical cancer—we observe both similar potential and parallel challenges. To ensure meaningful and sustainable adoption of AI in healthcare, it is essential to establish a comprehensive framework that addresses both the technical and broader operational challenges. While technical aspects such as generalizability, model explainability, and transparency are crucial, they represent only part of the equation. The more significant hurdles often lie in the operational and structural domains, which include regulatory concerns, infrastructural limitations, and socio-technical barriers.

For example, a recent white paper from the American Society of Cytopathology and other related organizations highlighted the importance of creating best practice guidelines for the integration of digital cytology and AI into clinical workflows. These guidelines are necessary to ensure that the technology is used effectively and safely across diverse settings, addressing regulatory and infrastructural gaps in AI adoption and helping to establish clear protocols and standards ([57]).

In addition a further study and a survey conducted by the American Society of Cytopathology explored the current landscape of digital cytology and AI in clinical settings [58,59]. The survey, which gathered responses from a broad range of professionals, highlighted the challenges of implementing AI, particularly regarding image quality, scanning speed, and the limited use of AI tools. It revealed that, while AI adoption in surgical pathology is more common, its integration into cytology practices remains limited. This survey provides valuable insights into the operational difficulties and areas where the AI framework needs to address infrastructural challenges.

Furthermore, addressing socio-technical barriers—such as the need for training and overcoming resistance from healthcare professionals—is crucial. The white paper recommends fostering collaboration between clinicians, data scientists, and public health authorities to build trust and support the integration of AI [57]. Additionally, training programs and initiatives that promote knowledge exchange are essential to ensure that professionals are well-equipped to use AI tools confidently.

Thus, a unifying framework for AI adoption must include both high-level strategic guidelines and context-specific actions. These actions, such as the surveys conducted in [59], help gather input from stakeholders, ensuring that the framework aligns with the real-world needs of healthcare providers. By addressing both technical and operational aspects, this framework can enable AI to be adopted meaningfully and sustainably, ensuring long-term improvements in healthcare delivery.

Regarding the opinion survey on AI integration in cytology, the importance of focusing on all the professional roles involved was emphasized. A study conducted through CAWI [60] allowed assessment of the perspectives of healthcare professionals on AI’s potential to enhance diagnostic accuracy and workflow efficiency, while also identifying barriers to adoption such as resistance to change, implementation costs, and concerns about AI reliability. Tailored training and continuous support were highlighted as key factors for successful AI integration into digital cytology workflows [60].

These studies [55,56,57,58,59,60], while not specifically focused on cervical cancer, still provide valuable insights into the integration of AI in cytology and cytopathology, particularly in addressing technical, operational, and socio-technical challenges. While AI applications in general settings are promising, there is a need for more targeted initiatives for cervical cancer, as seen in other fields. A collaborative effort among professionals and researchers could accelerate the sustainable and meaningful adoption of AI, addressing the specific challenges of this oncological area. Additionally, since artificial intelligence is continuously evolving, it is essential to develop dynamic frameworks that can adapt to new discoveries and technologies, ensuring responsible and beneficial AI integration into clinical practices.

### 4.3. Innovative AI and Automated Platforms for Cervical Cancer Screening and Diagnosis

While early efforts to enhance cervical cancer screening with computational tools date back over two decades—such as the Hologic ThinPrep Imaging System and the BD FocalPoint Slide Profiler, which offered computer-assisted screening with automated slide scanning and cell pre-classification—these systems did not employ modern AI or deep learning models. Rather, they relied on rule-based image analysis and pattern recognition to assist cytotechnologists. These pioneering technologies were instrumental in initiating the shift from manual to semi-automated cytology, laying the groundwork for the AI-integrated platforms emerging in recent years. Given the rapid evolution of machine learning and deep neural networks, this overview focuses intentionally on commercially available systems developed or clinically introduced in the past five years, which represent a new generation of AI-powered tools with enhanced analytical capacity, scalability, and regulatory momentum.

The landscape of artificial intelligence (AI) applications in cervical cancer diagnostics includes today a variety of commercial tools that either focus specifically on cervical cytology or provide broader cytology and pathology solutions adaptable to cervical cancer screening, but also colposcopy. All these platforms utilize AI to enhance diagnostic accuracy, efficiency, and reproducibility by automating image analysis, improving sample quality, or supporting clinical decision-making.

It is important to note that, alongside emerging AI-native tools, there are also more established, historically developed platforms that are not purely AI-based but have played a crucial role in automating cytology workflows. These earlier systems often rely on rule-based algorithms and image processing techniques, providing important foundations upon which current AI innovations can build.

This overview is not intended to identify a “best of the bunch,” but rather to present representative examples illustrating the diversity and potential of AI integration in this critical healthcare area.

Devices specifically designed for cervical cancer detection with integrated AI technologies include Cerviray AI by Cerviray from South Korea using colposcopy images. This platform employs deep learning and convolutional neural networks to analyze images. Another example is CellPrep Plus from Biodyne, also based in South Korea. While primarily a liquid-based cytology processor focused on preparing high-quality cervical samples, it integrates AI tools to enhance the quality of cytological slides, thereby supporting subsequent AI-driven analyses or manual cytological review. This device emphasizes optimizing specimen quality as a foundation for reliable cytology diagnostics.

The Genius™ Digital Diagnostics System from Hologic in the USA is a sophisticated AI-powered cytology platform cleared by the FDA specifically for cervical cancer screening. Combining advanced volumetric imaging with AI algorithms, it automates Pap smear slide analysis, identifies abnormal cervical cells, and helps cytotechnologists focus on suspicious regions to improve workflow efficiency and diagnostic precision. Similarly, France-based Datexim offers the CytoProcessor^®^, an AI-enabled cytology diagnostic solution employing machine learning to assist pathologists in detecting cervical abnormalities. This CE-marked device aims to streamline cervical cancer screening workflows while maintaining high sensitivity. The ScanAI platform from Scanome, USA, integrates AI and machine learning to deliver automated image analysis, feature extraction, and risk stratification tailored for cervical cytology diagnostics, enhancing diagnostic throughput and consistency in both clinical and research settings. MobileODT’s EVA^®^ Digital Colposcope from Israel focuses on cervical imaging rather than cytology, but its inclusion is justified by its integration of AI-based decision support to aid clinicians in detecting precancerous changes during point-of-care examinations. While not a cytology device per se, it exemplifies how AI is being translated into practical tools across different stages of the cervical cancer screening continuum. Its relevance is particularly pronounced in low-resource settings, where scalable, real-time visual assessment can complement or substitute cytological analysis when laboratory infrastructure is limited.

There are also versatile platforms initially developed for general cytology or pathology applications but adaptable and applicable to cervical cytology diagnostics. The ThinPrep Imaging System by Hologic (USA) is a well-established automated cytology imaging platform widely used for various specimen types, including cervical Pap smears. Though not purely AI-based, it combines automated slide scanning and algorithmic analysis to pre-screen slides and highlight suspicious cells for further review, substantially improving screening efficiency. Similarly, the BD FocalPoint Slide Profiler (USA) employs algorithmic profiling to pre-screen Pap smear slides, ranking them by likelihood of abnormality. While not leveraging deep learning, the system uses rule-based algorithms and image pattern recognition to prioritize cases that require cytotechnologist review, thus enhancing both productivity and diagnostic consistency. These technologies, though grounded in earlier generations of AI and automation, laid the groundwork for current machine learning–driven solutions and continue to serve as robust tools in cervical cancer screening workflows.

Paige AI, a USA-based company, develops deep learning models primarily targeting digital pathology across multiple cancers. Their adaptable platform supports cytology applications, including cervical samples, aiding pathologists in malignant cell identification to enhance diagnostic accuracy and lab workflow. OptraSCAN’s CytoSiA is an AI-powered digital pathology solution that automates image analysis and quality control for diverse cytology specimens, including cervical samples. By providing detailed cell-level assessments, it reduces manual workload and improves the detection of abnormalities.

Together, these AI-driven platforms reflect the current spectrum of tools available for cervical cancer diagnostics. Some are finely tuned for cervical cytology workflows, employing specialized AI models and sample processing techniques, while others offer broader pathology applications with adaptability to cervical cancer screening. This overview provides a representative sample rather than a comprehensive ranking, highlighting the varied approaches and technological innovations that AI brings to the early detection and management of cervical cancer.

Table 2 reports a sketch of the overviewed devices.

### 4.4. Limitations

This narrative review has a few limitations that should be noted. Firstly, the focus on studies published in the last five years allowed the review to concentrate on current trends and technological advancements. This decision, while aligned with the fast-paced evolution of artificial intelligence in healthcare, may have led to the exclusion of earlier foundational studies that still hold relevance. Secondly, the use of PubMed and Scopus as primary databases may have missed relevant studies published in non-English languages or indexed in regional or less widely covered repositories. Expanding the search to include additional sources—such as Embase, Web of Science, or pre-print platforms—could offer a broader and more representative picture of global research efforts. Although not peer-reviewed, preprints may represent an important source of innovation in this rapidly evolving domain and could be cautiously explored to identify early signals of technological and clinical shifts. For example, we excluded [71], where the authors present Cytoreader-V2, an AI-based tool designed to improve the accuracy, reproducibility, and scalability of dual-stain cytology (p16/Ki67) for cervical cancer screening, aiming to enhance its global implementation in digital pathology.

Thirdly, the review focused solely on studies that applied AI directly to clinical or preclinical screening settings. While this ensured a practical and translational perspective, it may have overlooked conceptual, ethical, or algorithmic contributions that, though not yet validated in clinical environments, could substantially influence future development trajectories.

Lastly, the narrative format itself entails certain trade-offs. Unlike systematic reviews, narrative reviews do not follow rigid inclusion protocols or formal quality appraisal frameworks, which can introduce a degree of subjectivity in study selection and interpretation. This could lead to potential biases, including selection bias, confirmation bias, and overrepresentation of studies from well-resourced geographic or institutional settings. We acknowledge these limitations, yet we argue that in emerging and highly multidisciplinary domains like AI in cervical cancer screening, a narrative approach offers strategic advantages.

The narrative format enables the integration of heterogeneous evidence sources, conceptual mapping across disciplines, and identification of underexplored but potentially transformative themes. In contrast to the procedural rigor of systematic reviews—which may struggle to capture innovation still in flux—narrative reviews allow for interpretive synthesis and horizon scanning. This is particularly relevant in contexts where the field is characterized by rapid technological shifts, evolving evaluation standards, and varying levels of clinical readiness.

Rather than presenting an alternative to systematic reviews, our aim is to complement them. We offer a timely, concept-driven overview that can inform, contextualize, and inspire future systematic efforts. In this perspective, a hybrid approach—combining the narrative lens with methodological rigor—may represent the most productive strategy for evidence synthesis in complex and evolving fields such as this.

## 5. Bridging the Gap: AI as the Future of Cervical Cancer Cytology

Despite decades of progress, cervical cytology remains challenged by critical limitations that affect the overall effectiveness of cervical cancer screening programs worldwide.

*First*, the subjectivity of cytological interpretation continues to be a major constraint. The evaluation of Pap smears depends heavily on the visual expertise of cytopathologists and trained screeners, which can introduce considerable inter- and intra-observer variability. This variability may lead to false negatives—delaying timely diagnosis—or false positives, resulting in unnecessary anxiety and follow-up procedures.

*Second*, the sheer volume of cytology samples, especially in large-scale or resource-constrained settings, places a burden on laboratories and human resources. Screening fatigue and logistical pressure can contribute to diagnostic errors and reduce overall efficiency.

*Third*, borderline or ambiguous findings often require reflex testing (e.g., HPV genotyping) or additional diagnostic steps such as colposcopy-guided biopsy. These multi-tiered workflows may delay clinical decisions, add costs, and increase the risk of loss to follow-up.

*Fourth*, although HPV testing has complemented cytology, integrating these diagnostic modalities remains complex. High-risk HPV positivity does not always indicate cytological abnormalities or progression to cancer, and requires contextual interpretation, which adds layers of diagnostic uncertainty.

*Finally*, variability in sample collection, preparation (e.g., conventional vs. liquid-based cytology), and reporting standards continues to impact diagnostic reproducibility across laboratories, limiting the comparability and scalability of screening programs.

In this context, AI-assisted diagnostic tools offer a promising path forward. By enhancing consistency, reducing human error, and supporting real-time decision-making, AI has the potential to transform cervical cancer cytology. These technologies can optimize workload, support standardization, and ultimately contribute to earlier detection and better outcomes. As the evidence base grows, the integration of AI into cervical cytology workflows may prove essential to bridging gaps in access, quality, and precision—bringing us closer to the global elimination of cervical cancer as a public health threat.

## 6. Conclusions

Artificial intelligence (AI) has demonstrated considerable promise in revolutionizing the field of cervical cancer diagnostics, specifically in cytology. As evidenced by the rapid growth of AI-related research and its increasing adoption in clinical practices, this technology is poised to enhance diagnostic accuracy, efficiency, and personalization in cancer care. However, despite the promising opportunities AI presents, several key challenges must be addressed to ensure its successful integration into healthcare systems worldwide.

A notable contribution of this narrative review is its ability to provide a comprehensive overview of both the potential benefits and challenges associated with AI in cervical cancer diagnostics. The review emphasizes the transformative power of AI to automate and standardize cytological interpretation, improve triage and risk stratification, and enable more personalized and precise treatment strategies. Furthermore, AI’s potential to bridge disparities in healthcare access, particularly in low-resource settings, is a critical advantage, offering real-time decision support and reducing geographical and workforce gaps in healthcare delivery.

Despite these opportunities, significant barriers remain, including concerns about the generalizability of AI models, the lack of model explainability and transparency, and regulatory challenges. The generalization of AI algorithms to diverse patient populations and clinical environments remains a critical hurdle, as does the need for transparency in AI decision-making processes. Additionally, regulatory and infrastructural challenges, such as data privacy concerns and the absence of standardized clinical guidelines, must be addressed to ensure safe and widespread adoption.

The integration of AI into cervical cancer diagnostics is also constrained by socio-technical barriers, including resistance from healthcare professionals, limited training, and the need for significant shifts in clinical workflows. These challenges underscore the necessity for coordinated efforts across multiple sectors, including healthcare providers, researchers, data scientists, and public health authorities, to create a unified framework that supports the sustainable integration of AI.

Looking forward, the rapid expansion of AI applications in cervical cancer diagnostics, coupled with its promising market growth, suggests that AI has the potential to transform cancer care globally. However, this transformation will require addressing both technical and operational challenges through comprehensive strategies and targeted initiatives. By fostering collaboration, creating clear guidelines, and investing in education and training, the healthcare community can ensure that AI’s integration leads to improved patient outcomes and more effective healthcare delivery.

Ultimately, this narrative review contributes to the ongoing dialogue surrounding AI in cervical cancer diagnostics by highlighting the complex interplay of opportunities and challenges. It calls for continued research and collaborative efforts to overcome these hurdles and realize the full potential of AI in improving cancer diagnostics and treatment.

## Figures and Tables

**Table 1:** Emerging themes in focus.

Reference	Brief Summary	Focus	Application of AI	Details on AI
Xue et al. (2025) [21]	This study presents a deep learning-based model for detecting cervical cancer and precancerous lesions from liquid-based cytology samples. The AI model outperforms traditional methods in accuracy, sensitivity, and specificity, leading to better early detection of cervical cancer.	AI in cervical cancer detection	The AI model automates the detection process, enabling more accurate and faster identification of cervical cancer at early stages, improving diagnostic outcomes.	Deep learning model applied to whole-slide liquid-based cytology images for detection and triage. Validated on >28,000 cases across nine hospitals, showing improved sensitivity and reduced reading time vs. cytopathologists.
Taghados et al. (2025) [22]	The paper introduces CausalCervixNet, a deep learning model designed to classify cervical cancer cells using a causal inference framework. By leveraging causal insights, the model improves the interpretability and accuracy of cervical cancer diagnoses, offering a new dimension for AI in medical imaging.	Deep learning in cervical cell classification	The AI-driven model provides more accurate and interpretable classifications of cervical cancer cells, enhancing clinicians’ diagnostic accuracy and treatment decisions.	CausalCervixNet employs a convolutional neural network architecture enhanced with causal inference techniques to model and identify underlying causal relationships in cervical cell images, moving beyond traditional correlation-based DL models. This integration allows the AI to provide more interpretable and explainable diagnostic outputs by uncovering latent causal factors that drive accurate cell classification.
Himabindu et al. (2025) [23]	The study introduces a hybrid deep learning model that integrates a Swin Transformer for analyzing colposcopy images. The model aims to detect cervical cancer and precancerous lesions with high accuracy, providing an automated tool to assist in the interpretation of colposcopic images, discussing their complementary role beyond cytology.	AI in colposcopy image analysis as a complimentary solution beyond the cytology	AI enhances the accuracy of cervical cancer detection by automating the analysis of colposcopy images, allowing clinicians to detect abnormalities that might be overlooked in manual examination.	The AI system uses Wiener filtering for image denoising, followed by a Swin Transformer to extract detailed features from colposcopy images. It combines three deep learning models—autoencoder, bidirectional GRU, and deep belief network—in an ensemble to improve classification accuracy. Hyperparameters are optimized via the Pelican Optimization Algorithm. This integrated approach achieves 99.44% accuracy in cervical cancer screening.
Yang et al. (2025) [24]	This paper identifies a hypermethylation marker (ZSCAN18) for cervical cancer and combines it with an AI-driven diagnostic model. By integrating molecular biomarkers with imaging data, the model aims to improve diagnostic precision and identify high-risk patients for early intervention.	Biomarker-based diagnostics	AI integrates molecular biomarkers like ZSCAN18 with traditional cytological methods to create a more accurate diagnostic tool for cervical cancer, aiding in early detection and personalized treatment.	This study integrated machine learning with bioinformatics to analyze methylation and gene expression data from large cervical cancer cohorts. Using feature selection and ridge regression, a diagnostic model was developed based on methylation biomarkers, notably in the ZSCAN18 promoter region. The model achieved high diagnostic accuracy (AUC 0.9421) in validation. Machine learning enabled effective identification and prioritization of methylation markers correlated with disease severity, supporting both diagnosis and potential therapeutic targeting.
Dong et al. (2025) [25]	The research presents SMART-HPV, a machine learning model for HPV genotyping that aims to enhance risk stratification in cervical cancer screening. By analyzing multiple factors, the AI model helps identify patients at higher risk for cervical cancer, improving the efficiency of screening protocols.	AI for risk stratification in HPV testing	SMART-HPV uses AI to analyze HPV genotyping data, allowing for more accurate identification of high-risk individuals and ensuring that resources are focused on patients who require immediate attention.	This study developed and validated four machine learning models—XGBoost, SVM, Random Forest, and Naïve Bayes—using hrHPV full genotyping data from over 1.1 million women to predict cervical precancerous lesions (CIN2+ and CIN3+). The optimized XGBoost model demonstrated high discrimination (AUROC up to 0.989) and reliable calibration across multiple large external validation cohorts. It incorporated clinical features and HPV genotypes to stratify risk and guide colposcopy referrals, especially useful in low screening rate settings. Decision curve analysis confirmed its clinical utility for improving referral decisions.
Pang et al. (2024) [26]	This study focuses on using AI to correct mislabeled cervical pathology images and accurately detect abnormal cell clusters. By improving image labeling accuracy, the AI system increases the reliability of automated cervical cancer screenings, particularly in large-scale public health settings.	Image correction and cell group detection	AI addresses mislabeled pathology images and enhances the ability to detect abnormal cell groups, improving classification accuracy and contributing to more reliable cervical cancer diagnostics.	This study presents PGCC-Net, a deep learning network for cervical cell detection that integrates clinical prior knowledge and corrects ambiguous labels. The method decomposes detection into sub-tasks for grouping cells, improving learning of cell structures. To tackle label ambiguity caused by complex Bethesda system categories and pathologist variability, a label correction module uses feature similarity and cluster centers to refine annotations. Validated on public (7410 images) and private (13,526 images) datasets, PGCC-Net outperforms existing cervical cell detection methods, enhancing diagnostic accuracy and efficiency.
Zhang et al. (2025) [27]	This research develops a dataset of annotated cervical cytology images to train AI models for cervical cancer screening. The dataset enables the creation of more accurate AI models that can automate the screening process, providing more reliable results for early-stage cancer detection.	AI model training using annotated datasets	AI leverages large, annotated datasets to train models for cervical cancer screening, which in turn improves the accuracy of automated diagnostic tools and speeds up the screening process.	The study implements deep learning-based models trained on a large annotated dataset of 8037 cervical cytology images from ThinPrep slides to detect abnormal cervical cells. The models leverage convolutional neural networks (CNNs) optimized for cytology image features to improve detection accuracy and robustness despite limited prior datasets.
Chun et al. (2024) [28]	This paper explores the use of contrastive self-supervised learning to automate the screening of precancerous cervical cells. The method allows AI models to learn from large datasets without the need for labeled images, making it a cost-effective solution for large-scale screening efforts.	Self-supervised learning in cancer detection	AI uses self-supervised learning to detect precancerous cells in cervical cancer screening, providing an efficient, cost-effective alternative to traditional manual methods.	The AI framework leverages contrastive self-supervised learning (SSL) enhanced with color distribution augmentations to train the model solely on normal cervical cell images, enabling it to learn robust feature representations without requiring extensive abnormal cell annotations. This distribution-augmented SSL approach enhances the model’s sensitivity to subtle morphological and color variations typical of high-grade lesions (HSIL and ASC-H), while kernel density estimation (KDE) is used post-training to model and detect deviations in cell distributions, enabling precise abnormality detection despite variability in staining and imaging conditions.
Pang et al. (2025) [29]	The study discusses the application of AI for correcting label credibility errors in cervical cancer screening images. By addressing inconsistencies in image labeling, the AI model improves the overall accuracy and reliability of cervical cancer diagnostic systems.	Image labeling improvement for classification	AI enhances classification accuracy by correcting label errors in cytology images, which contributes to more reliable cervical cancer diagnoses, reducing human error in the screening process.	The method uses contrastive learning to extract discriminative features and applies unsupervised clustering to correct noisy labels in cervical cell images. Label credibility is analyzed by comparing cluster features to class centers, improving multi-class classification stability via a synergistic grouping loss with momentum. This approach achieved over 92% accuracy on a large, multi-hospital dataset.
Welch et al. (2024) [30]	This paper introduces a cross-validated cervical cytology dataset for training AI models aimed at improving cervical cancer detection. By providing a standardized set of images, the dataset allows AI systems to achieve higher levels of accuracy and consistency in screening.	Dataset development for AI model training	AI models trained on high-quality, cross-validated datasets improve cervical cancer detection by offering more reliable and standardized results across different diagnostic settings.	The BMT dataset supports AI training by providing multicellular ThinPrep^®^ images that reflect real screening variability. AI models trained on this dataset can better handle visual differences from varying slide preparations, improving generalization. This enables more accurate classification of cervical cell abnormalities in diverse clinical settings.
Lim et al. (2024) [31]	The research integrates microbiome analysis with liquid-based cytology to enhance cervical cancer diagnostics. By incorporating microbiome data into AI-driven models, the study aims to identify new biomarkers that could improve the accuracy of cervical cancer screening.	Microbiome and AI integration	AI models combine microbiome data with cytology results to enhance cervical cancer detection, offering more precise diagnostic capabilities and uncovering new biomarkers for early-stage diagnosis.	Supervised machine learning was applied to microbiome data from LBC and SWAB samples to distinguish sampling methods based on microbial features. The AI analysis highlighted challenges in classification due to high similarity between sample types. Network analysis and weighted co-expression methods further demonstrated weak correlations between microbial clusters, sampling techniques, and clinical data, underscoring the robustness of microbiome profiles across methods and the potential for integrated diagnostic approaches.
Muksimova et al. (2024) [32]	This study explores the use of reinforcement learning for classifying cervical cancer images. The model adapts itself based on feedback, improving its diagnostic performance over time and offering a more flexible approach to cervical cancer detection.	Reinforcement learning in cervical cancer diagnosis	AI models powered by reinforcement learning dynamically improve their ability to classify cervical cancer images, allowing for more adaptive and accurate detection as they process more data.	The proposed RL-CancerNet model combines EfficientNetV2 and Vision Transformers within a reinforcement learning framework to enhance cervical cancer diagnosis. EfficientNetV2 extracts local features, while ViTs capture global image dependencies. A reinforcement learning agent dynamically mitigates class imbalance by focusing on minority classes. A Supporter Module with Conv3D, BiLSTM, and attention mechanisms further improves contextual feature learning. Evaluated on benchmark datasets, RL-CancerNet achieved 99.7% accuracy, outperforming state-of-the-art methods and demonstrating robust detection of subtle diagnostic patterns in cervical cytology images.
Hays (2024) [33]	This review examines the growing role of AI in cytopathology, focusing on its application in cervical cancer detection. The paper highlights the advancements in AI systems that offer higher accuracy and faster diagnosis compared to traditional methods.	AI in cytopathology	AI improves the accuracy and efficiency of cytopathological diagnoses, including cervical cancer, by automating the interpretation of complex cytological images.	This review emphasizes the role of AI—especially machine learning and deep learning—in cytopathological diagnosis. AI models extract features from cytology images, improving classification accuracy, sensitivity, and specificity in detecting cervical and other cancers. These models reduce human error and interobserver variability by learning from large labeled datasets. Both standalone AI and human-AI hybrid approaches show promising diagnostic performance. The study highlights the increasing deployment of AI-assisted tools in clinical cytopathology to enhance diagnostic precision and workflow efficiency.
Goldstein et al. (2024) [34]	This article discusses the future of cervical cancer screening with AI, outlining its potential to reduce false negatives and improve early detection. The paper predicts that AI-driven technologies will become integral to cervical cancer prevention efforts worldwide.	Future trends in cervical cancer screening	AI is expected to transform cervical cancer screening by providing more accurate, cost-effective, and accessible diagnostic solutions, which could ultimately reduce cervical cancer incidence rates globally.	This study emphasizes AI applications in cervical cancer screening using deep learning architectures for image analysis. AI models utilize convolutional neural networks (CNNs) to extract detailed features from digital colposcopy and cytology images. These models perform automated classification and segmentation of HPV-related lesions and cervical dysplasia. Advanced AI techniques, like transfer learning, attention mechanisms, and ensemble models, improve diagnostic accuracy and robustness. Additionally, AI algorithms integrate data from rapid HPV tests and methylation assays to enhance risk prediction and screening efficiency, enabling scalable and cost-effective deployment in resource-limited environments.
Zhang et al. (2024) [35]	This study explores machine learning techniques for risk-stratifying women with high-grade squamous intraepithelial lesions (HSIL) in cervical cancer screening. The AI model helps identify women at greater risk for progression to cervical cancer, guiding treatment decisions.	Machine learning in risk stratification	AI improves cervical cancer screening by using machine learning to identify patients with high-grade lesions who are at increased risk, enabling personalized and timely interventions.	The study employed machine learning to predict pathological outcomes after conization in cervical HSIL patients. Among six models tested, the back propagation (BP) neural network demonstrated the highest predictive accuracy and robustness, outperforming logistic regression with an AUC of 0.91 on external validation. This AI model integrates multiple clinical and pathological variables to stratify patient risk effectively. A web-based tool based on the BP neural network was developed to support clinical decision-making, showcasing AI’s potential in enhancing personalized cervical cancer management.
Liu et al. (2024) [36]	This paper presents an AI-assisted film reading system combined with liquid-based cytology for cervical cancer screening. The system helps clinicians review and analyze cytology slides with greater speed and accuracy, improving the quality of screening programs.	AI and liquid-based cytology	AI-driven film reading systems enhance cervical cancer detection by automating the analysis of cytology slides, reducing human error and speeding up the screening process.	Combines automated image analysis with liquid-based cytology examination. The system uses AI algorithms for automated interpretation of cervical cytology images, improving detection accuracy through integration of image processing and pattern recognition.
Sood et al. (2024) [37]	The study applies transfer learning and attention mechanisms in AI to improve the detection of cervical abnormalities in Pap smears. This approach allows AI to focus on the most relevant features of the image, increasing diagnostic precision.	AI for Pap smear analysis	AI helps detect cervical abnormalities by applying advanced machine learning techniques, offering enhanced diagnostic accuracy for Pap smear analysis and early cancer detection.	Uses pre-trained convolutional neural networks (CNNs) fine-tuned with pap smear image data (transfer learning) to leverage learned features from large datasets. Attention mechanisms are integrated to enhance focus on relevant image regions, improving detection of cervical abnormalities.
Wu et al. (2024) [38]	This research investigates how AI can enhance both detection and triage in cervical cancer screening. The model identifies abnormalities in screening images and helps prioritize cases for further examination or biopsy, improving overall clinical workflow.	Enhancing screening with AI	AI improves cervical cancer screening by automating detection and triaging patients based on the severity of abnormalities, leading to quicker and more accurate diagnoses.	Discusses various AI approaches, such as machine learning classifiers, deep learning (including CNNs), and ensemble learning applied to image-based cervical cancer screening. Emphasizes current AI frameworks that support image analysis, risk stratification, and prediction for screening optimization.
Song et al. (2025) [39]	The study applies machine learning techniques to evaluate risk and efficacy in cervical cancer screening. By analyzing a wide range of patient data, the AI model helps determine the most effective screening protocols for different risk groups.	Risk stratification with machine learning	AI assists in risk assessment for cervical cancer by analyzing patient data and determining personalized screening and treatment protocols, improving clinical outcomes.	The study applies classical machine learning models (Random Forest, SVM) to integrate clinical and molecular data for risk stratification in cervical screening. Emphasis on model interpretability and validation with a large HPV cohort enhances clinical utility.
Qin et al. (2024) [40]	This research presents a comparative learning method to classify cervical cytology images using whole-slide scanning technology. The model aims to improve the detection of abnormal cells with high sensitivity and specificity.	Whole-slide image classification	AI enhances the detection of cervical cancer by analyzing whole-slide images, which provides a more comprehensive view of cytological samples for more accurate diagnoses.	Uses comparative deep learning on whole slide cytology images, employing convolutional neural networks with attention modules to distinguish normal vs. abnormal cells. Improves feature learning from large, high-resolution datasets.
Pereira et al. (2024) [41]	This study discusses the development of an AI-enhanced PCR method for HPV genotyping, which improves the accuracy of cervical cancer risk detection. The model helps identify high-risk HPV strains that are most likely to lead to cancer.	AI-driven HPV genotyping	AI models applied to HPV genotyping improve the accuracy and speed of cervical cancer risk assessment by identifying high-risk HPV types, enabling early intervention.	AI automates multiplex qPCR data analysis by processing fluorescence signals and classifying HPV genotypes. Machine learning models increase sensitivity and specificity, supporting scalable molecular diagnostics.
Ando et al. (2024) [42]	The paper presents an AI model for interpreting cell images and scoring abnormalities in Pap smears, aiming to automate and standardize the screening process for cervical cancer.	AI for image interpretation and scoring	AI automates the scoring of cervical cell abnormalities in Pap smears, improving the efficiency and consistency of cervical cancer screening, reducing the workload for pathologists.	This study proposes an interpretable AI framework for cervical cancer screening by developing cell image representations and abnormality scoring based on Pap smear images. The model emphasizes explainability, helping pathologists understand AI-driven decisions and improving trust in automated screening.
Cai et al. (2024) [43]	The HiCervix dataset is introduced, a comprehensive collection of cervical cytology images for AI model training. The dataset includes various types of abnormal cells, providing a robust resource for improving AI-based screening tools.	Large dataset for AI classification	AI models trained on the HiCervix dataset improve cervical cancer detection by offering higher accuracy in classifying cytological abnormalities, supporting early diagnosis.	HiCervix introduces a large, hierarchical dataset and benchmark for cervical cytology classification using deep learning. The paper presents state-of-the-art convolutional neural networks trained on multi-level annotations to enhance accuracy and robustness in cell-level classification.
Wang et al. (2024) [44]	AI is applied to develop a precision diagnostic tool that grades cervical cytology images and stages cervical cancer. The model helps clinicians identify cancer stages more accurately, improving treatment planning.	Precision diagnosis with AI	AI models enhance the staging of cervical cancer by accurately grading cytology images, supporting better treatment decisions and personalized care.	This paper describes a precision AI system that diagnoses cervical cytology grades and cancer with high accuracy. The model leverages ensemble deep learning architectures to improve diagnostic performance and supports clinical decision-making by providing detailed grading.
Mahajan et al. (2024) [45]	The study uses AI-enhanced deep learning techniques to improve cervical cancer classification in Pap smear images. The model achieves high accuracy, providing an automated and reliable tool for clinical diagnosis.	AI for improved Pap smear analysis	AI automates the analysis of Pap smear images, improving classification accuracy and providing clinicians with a more reliable tool for cervical cancer screening.	The authors enhance cervical cancer classification in Pap smear images through advanced segmentation techniques combined with deep progressive learning. Their AI pipeline improves feature extraction and classification accuracy by iteratively refining the model’s learning process.
Civit-Masot et al. (2024) [46]	This paper introduces a lightweight explainable AI (xAI) model for cervical cancer classification, offering transparency in decision-making. The model provides clinicians with understandable reasons for predictions, enhancing trust in AI-based diagnostic systems.	Explainable AI in cervical cancer detection	AI offers transparency in its decision-making process, which helps clinicians trust AI-based systems for cervical cancer detection, making them more likely to adopt these technologies in clinical practice.	This work presents a lightweight explainable AI (xAI) approach tailored for cervical cancer classification. The model balances computational efficiency and interpretability, enabling deployment in low-resource settings while providing insights into the AI decision process.
Zhang et al. (2024) [47]	The study develops a semi-supervised learning approach called SCAC for detecting abnormal cervical cells in cytology images. This model allows for accurate detection with limited labeled data, making it an efficient tool for large-scale screenings.	Semi-supervised learning for abnormal cell detection	AI uses semi-supervised learning to improve cervical cancer detection in cytology images, achieving high accuracy with fewer labeled data and making it scalable for large datasets.	SCAC proposes a semi-supervised learning algorithm for detecting cervical abnormal cells. By leveraging both labeled and unlabeled data, the method reduces annotation needs while maintaining high detection sensitivity and specificity.
Tang et al. (2023) [48]	The study applies AI-based ConvNeXt models to classify cervical precancerous lesions, offering enhanced detection accuracy. This approach allows for better identification of early-stage cervical abnormalities.	AI-based classification of precancerous lesions	AI models like ConvNeXt improve the classification of cervical lesions, leading to earlier detection and better patient outcomes.	The study uses ConvNeXt, a cutting-edge convolutional neural network, for high-precision classification of cervical precancerous lesions. The model achieves superior performance by integrating advanced feature extraction with fine-tuned network architectures.
Stegmüller et al. (2024) [49]	This research investigates the use of AI to triage HPV-positive women in low-resource settings, aiding in cervical cancer screening. The system prioritizes women based on risk, providing quicker and more efficient screenings.	Self-supervised learning for triage in low-resource settings	AI triages HPV-positive women in low-resource settings, improving access to cervical cancer screening by prioritizing high-risk cases for further diagnostic testing.	This paper develops a self-supervised learning framework for cervical cytology triage, designed for HPV-positive women in low-resource settings. The approach effectively learns from limited labeled data, improving screening accessibility and accuracy where data are scarce.
Rutili de Lima et al. (2024) [50]	This study proposes a deep learning approach using Mask RCNN for detecting and segmenting cervical cancer cells in tissue images. It achieves high detection accuracy and generates automatic reports for medical consultants.	Cervical cancer detection and segmentation	The AI model uses a Mask RCNN-based deep learning architecture to detect and segment cervical cancer cells, automatically generating reports to help medical professionals identify cancerous areas efficiently.	The authors apply Mask Region-based Convolutional Neural Networks (Mask R-CNN) to diagnose cervical cancer progression from Pap smear images. The method excels in precise localization and classification of lesion regions, aiding detailed pathological assessment.

**Table 2:** Sketch of the overviewed devices.

Device Name	Manufacturer	Focus Area	Reference
Cerviray AI	Cerviray (Seoul, Republic of Korea)	Colposcopy +AI	[61]
CellPrep Plus	Biodyne (Seoul, Republic of Korea)	Cervical cytology	[62]
ThinPrep Imaging System	Hologic (Marlborough, MA, USA)	General cytology, applicable to cervical	[63]
Genius™ Digital Diagnostics System	Hologic (Marlborough, MA, USA)	Cervical cytology + AI	[64]
CytoProcessor^®^	Datexim (Caen, France)	Cervical cytology + AI	[65]
ScanAI	Scanome (Warszawa, Poland )	Cervical cytology + AI	[66]
EVA^®^ Digital Colposcope	MobileODT (Tel Aviv, Israel)	Colposcopy + AI	[67]
Paige AI	Paige AI (New York, NY, USA)	General cytology/pathology	[68]
CytoSiA	OptraSCAN (San Jose, CA, USA)	General cytology + AI	[69]
BD FocalPoint Slide Profiler	BD (Franklin Lakes, NJ, USA)	Cervical cytology + algorithmic support	[70]

## Data Availability

Data are contained within the article.

## References

[1] Safaeian M., Solomon D., Castle P.E. (2007). Cervical cancer prevention–cervical screening: Science in evolution. Obstet. Gynecol. Clin. N. Am..

[2] Cytology Automation: An Overview. https://scispace.com/pdf/cytology-automation-an-overview-efultv68k5.pdf.

[3] Cooper D.B., Dunton C.J. (2025). Colposcopy. StatPearls.

[4] Pangarkar M.A. (2022). The Bethesda System for reporting cervical cytology. Cytojournal.

[5] Cox J.T. (2009). History of the use of HPV testing in cervical screening and in the management of abnormal cervical screening results. J. Clin. Virol..

[6] Bedell S.L., Goldstein L.S., Goldstein A.R., Goldstein A.T. (2020). Cervical Cancer Screening: Past, Present, and Future. Sex. Med. Rev..

[7] Sturgis C.D., Isoe C., McNeal N.E., Yu G.H., DeFrias D.V. (1998). PAPNET computer-aided rescreening for detection of benign and malignant glandular elements in cervicovaginal smears: A review of 61 cases. Diagn. Cytopathol..

[8] Saraiya M., Steben M., Watson M., Markowitz L. (2013). Evolution of cervical cancer screening and prevention in United States and Canada: Implications for public health practitioners and clinicians. Prev. Med..

[9] Saini T., Bansal B., Dey P. (2023). Digital cytology: Current status and future prospects. Diagn. Cytopathol..

[10] Yamashiro K., Kawamura N., Matsubayashi S., Dota K., Suzuki H., Mizushima H., Wakao F., Azumi N. (2004). Telecytology in Hokkaido Island, Japan: Results of primary telecytodiagnosis of routine cases. Cytopathology.

[11] Harangi B., Bogacsovics G., Toth J., Kovacs I., Dani E., Hajdu A. (2024). Pixel-wise segmentation of cells in digitized Pap smear images. Sci. Data.

[12] Cervical Cancer Screening (PDQ®)–Health Professional Version. https://www.cancer.gov/types/cervical/hp/cervical-screening-pdq.

[13] Bao H., Bi H., Zhang X., Zhao Y., Dong Y., Luo X., Zhou D., You Z., Wu Y., Liu Z. (2020). Artificial intelligence-assisted cytology for detection of cervical intraepithelial neoplasia or invasive cancer: A multicenter, clinical-based, observational study. Gynecol. Oncol..

[14] Liu L., Liu J., Su Q., Chu Y., Xia H., Xu R. (2024). Performance of artificial intelligence for diagnosing cervical intraepithelial neoplasia and cervical cancer: A systematic review and meta-analysis. EClinicalMedicine.

[15] Wang J., Wang T., Han R., Shi D., Chen B. (2025). Artificial intelligence in cancer pathology: Applications, challenges, and future directions. Cytojournal.

[16] Ouh Y.-T., Kim T.J., Ju W., Kim S.W., Jeon S., Kim S.-N., Kim K.G., Lee J.-K. (2024). Development and validation of artificial intelligence-based analysis software to support screening system of cervical intraepithelial neoplasia. Sci. Rep..

[17] Dellino M., Cerbone M., d’Amati A., Bochicchio M., Laganà A.S., Etrusco A., Malvasi A., Vitagliano A., Pinto V., Cicinelli E. (2024). Artificial Intelligence in Cervical Cancer Screening: Opportunities and Challenges. AI.

[18] Akazawa M., Hashimoto K. (2021). Artificial intelligence in gynecologic cancers: Current status and future challenges—A systematic review. Artif. Intell. Med..

[19] FDA Clears AI-Powered Digital Cytology Platform for Cervical Cancer. https://www.diagnosticimaging.com/view/fda-clears-ai-powered-digital-cytology-platform-for-cervical-cancer.

[20] Evaluation of Automatic Class III Designation for GeniusTM Digital Diagnostics System with the Genius TM Cervical Al Algorithm. https://www.accessdata.fda.gov/cdrh_docs/reviews/DEN210035.pdf.

[21] Xue P., Dang L., Kong L.H., Tang H.P., Xu H.M., Weng H.Y., Wang Z., Wei R.G., Xu L., Li H.X. (2025). Deep learning enabled liquid-based cytologmodel for cervical precancer and cancer detection. Nat. Commun..

[22] Taghados Z., Azimifar Z., Monsefi M., Jahromi M.A. (2025). CausalCervixNet: Convolutional neural networks with causal insight (CICNN) in cervical cancer cell classification-leveraging deep learning models for enhanced diagnostic accuracy. BMC Cancer.

[23] Himabindu D.D., Lydia E.L., Rajesh M.V., Ahmed M.A., Ishak M.K. (2025). Leveraging swin transformer with ensemble of deep learning model for cervical cancer screening using colposcopy images. Sci. Rep..

[24] Yang J., Chen S., Liu Y., Wang P., Zhao J., Yi J., Wei J., Wang R. (2025). Identification of a novel hypermethylation marker, ZSCAN18, and construction of a diagnostic model in cervical cancer. Clin. Transl. Oncol..

[25] Dong B., Lu Z., Yang T., Wang J., Zhang Y., Tuo X., Wang J., Lin S., Cai H., Cheng H. (2025). Development, validation, and clinical application of a machine learning model for risk stratification and management of cervical cancer screening based on full-genotyping hrHPV test (SMART-HPV): A modelling study. Lancet Reg. Health West. Pac..

[26] Pang W., Ma Y., Jiang H., Yu Q. (2024). Cells Grouping Detection and Confusing Labels Correction on Cervical Pathology Images. Bioengineering.

[27] Zhang X., Ji J., Zhang Q., Zheng X., Ge K., Hua M., Cao L., Wang L. (2025). A large annotated cervical cytology images dataset for AI models to aid cervical cancer screening. Sci. Data.

[28] Chun J., Yu A., Ko S., Chong G., Park J., Han H., Park N.J., Cho J. (2024). Automated Screening of Precancerous Cervical Cells Through Contrastive Self-Supervised Learning. Life.

[29] Pang W., Qiu Y., Jin S., Jiang H., Ma Y. (2025). Label credibility correction based on cell morphological differences for cervical cells classification. Sci. Rep..

[30] Welch E.C., Lu C., Sung C.J., Zhang C., Tripathi A., Ou J. (2024). BMT: A Cross-Validated ThinPrep Pap Cervical Cytology Dataset for Machine Learning Model Training and Validation. Sci. Data.

[31] Lim C., Seo Y.J., Lee J.Y., Jung E.S., Lee S., Kim H., Kim K., Kim J.M. (2024). Evaluation of the cervical liquid-based cytology sample as a microbiome resource for dual diagnosis. PLoS ONE.

[32] Muksimova S., Umirzakova S., Shoraimov K., Baltayev J., Cho Y.I. (2024). Novelty Classification Model Use in Reinforcement Learning for Cervical Cancer. Cancers.

[33] Hays P. (2024). Artificial intelligence in cytopathological applications for cancer: A review of accuracy and analytic validity. Eur. J. Med. Res..

[34] Goldstein A., Gersh M., Skovronsky G., Moss C. (2024). The Future of Cervical Cancer Screening. Int. J. Women’s Health.

[35] Zhang L., Tian P., Li B., Xu L., Qiu L., Bi Z., Chen L., Sui L. (2024). Risk-stratified management of cervical high-grade squamous intraepithelial lesion based on machine learning. J. Med. Virol..

[36] Liu D., Chu J. (2024). Analysis of effectiveness in an artificial intelligent film reading system combined with liquid based cytology examination for cervical cancer screening. Am. J. Transl. Res..

[37] Sood T., Khandnor P., Bhatia R. (2024). Enhancing pap smear image classification: Integrating transfer learning and attention mechanisms for improved detection of cervical abnormalities. Biomed. Phys. Eng. Express.

[38] Wu T., Lucas E., Zhao F., Basu P., Qiao Y. (2024). Artificial intelligence strengthens cervical cancer screening—Present and future. Cancer Biol. Med..

[39] Song H., Lee H.Y., Oh S.A., Seong J., Hur S.Y., Choi Y.J. (2025). Application of Machine Learning Algorithms for Risk Stratification and Efficacy Evaluation in Cervical Cancer Screening among the ASCUS/LSIL Population: Evidence from the Korean HPV Cohort Study. Cancer Res. Treat..

[40] Qin J., He Y., Liang Y., Kang L., Zhao J., Ding B. (2024). Cell comparative learning: A cervical cytopathology whole slide image classification method using normal and abnormal cells. Comput. Med. Imaging Graph..

[41] Pereira A.R., Redzic N., Van Vooren S., Pelak K., Broekmans A., Desloovere G., Vanden Broeck D., Kehoe K., Bogers J., Coppens A. (2024). Development, Validation, and Implementation of an Augmented Multiwell, Multitarget Quantitative PCR for the Analysis of Human Papillomavirus Genotyping through Software Automation, Data Science, and Artificial Intelligence. J. Mol. Diagn..

[42] Ando Y., Cho J., Park N.J., Ko S., Han H. (2024). Toward Interpretable Cell Image Representation and Abnormality Scoring for Cervical Cancer Screening Using Pap Smears. Bioengineering.

[43] Cai D., Chen J., Zhao J., Xue Y., Yang S., Yuan W., Feng M., Weng H., Liu S., Peng Y. (2024). HiCervix: An ExtensiveHierarchical Dataset and Benchmark for Cervical Cytology Classification. IEEE Trans. Med. Imaging.

[44] Wang J., Yu Y., Tan Y., Wan H., Zheng N., He Z., Mao L., Ren W., Chen K., Lin Z. (2024). Artificial intelligence enables precision diagnosis of cervical cytology grades and cervical cancer. Nat. Commun..

[45] Mahajan P., Kaur P. (2024). Improving cervical cancer classification in PAP smear images with enhanced segmentation and deep progressive learning-based techniques. Diagn. Cytopathol..

[46] Civit-Masot J., Luna-Perejon F., Muñoz-Saavedra L., Domínguez-Morales M., Civit A. (2024). A lightweight xAI approach to cervical cancer classification. Med. Biol. Eng. Comput..

[47] Zhang Z., Yao P., Chen M., Zeng L., Shao P., Shen S., Xu R.X. (2024). SCAC: A Semi-Supervised Learning Approach for Cervical Abnormal Cell Detection. IEEE J. Biomed. Health Inform..

[48] Tang J., Zhang T., Gong Z., Huang X. (2023). High Precision Cervical Precancerous Lesion Classification Method Based on ConvNeXt. Bioengineering.

[49] Stegmüller T., Abbet C., Bozorgtabar B., Clarke H., Petignat P., Vassilakos P., Thiran J.P. (2024). Self-supervised learning-based cervical cytology for the triage of HPV-positive women in resource-limited settings and low-data regime. Comput. Biol. Med..

[50] Rutili de Lima C., Khan S.G., Shah S.H., Ferri L. (2023). Mask region-based CNNs for cervical cancer progression diagnosis on pap smear examinations. Heliyon.

[51] Thakur N., Alam M.R., Abdul-Ghafar J., Chong Y. (2022). Recent Application of Artificial Intelligence in Non-Gynecological Cancer Cytopathology: A Systematic Review. Cancers.

[52] Cervical Cancer Screening Market. https://www.futuremarketinsights.com/reports/cervical-cancer-screening-market.

[53] Huang Q., Su W., Li S., Lin Y., Cheng Z., Chen Y., Mo Y. (2025). A bibliometric analysis of artificial intelligence applied to cervical cancer. Front. Med..

[54] Hou X., Shen G., Zhou L., Li Y., Wang T., Ma X. (2022). Artificial Intelligence in Cervical Cancer Screening and Diagnosis. Front. Oncol..

[55] Giansanti D. (2024). AI in Cytopathology: A Narrative Umbrella Review on Innovations, Challenges, and Future Directions. J. Clin. Med..

[56] Lastrucci A., Giarnieri E., Carico E., Giansanti D. (2024). Revolutionizing Cytology and Cytopathology with Natural Language Processing and Chatbot Technologies: A Narrative Review on Current Trends and Future Directions. Bioengineering.

[57] Kim D., Sundling K.E., Virk R., Thrall M.J., Alperstein S., Bui M.M., Chen-Yost H., Donnelly A.D., Lin O., Liu X. (2024). Digital cytology part 2: Artificial intelligence in cytology: A concept paper with review and recommendations from the American So-ciety of Cytopathology Digital Cytology Task Force. J. Am. Soc. Cytopathol..

[58] Kim D., Sundling K.E., Virk R., Thrall M.J., Alperstein S., Bui M.M., Chen-Yost H., Donnelly A.D., Lin O., Liu X. (2024). Digital cytology part 1: Digital cytology implementation for practice: A concept paper with review and recommendations from the American Society of Cytopathology Digital Cytology Task Force. J. Am. Soc. Cytopathol..

[59] Kim D., Thrall M.J., Michelow P., Schmitt F.C., Vielh P.R., Siddiqui M.T., Sundling K.E., Virk R., Alperstein S., Bui M.M. (2024). The Current State of Digital Cy-tology and Artificial Intelligence (AI): Global Survey Results from the American Society of Cytopathology Digital Cytology Task Force. J. Am. Soc. Cytopathol..

[60] Giansanti D., Carico E., Lastrucci A., Giarnieri E. (2025). Surveying the Digital Cytology Workflow in Italy: An Initial Report on AI Integration Across Key Professional Roles. Healthcare.

[61] Harsono A.B., Susiarno H., Suardi D., Mantilidewi K.I., Wibowo V.D., Hidayat Y.M. (2025). Results comparison of cervical cancer early detection using cerviray ^®^ with VIA test. BMC Res. Notes.

[62] CellPrep Plus. https://biodyne.en.ec21.com/CellPrep_Plus--8770069_8770070.html.

[63] ThinPrep® Operator’s Manual Imaging System Image Processor. https://www.hologic.com/sites/default/files/2017-12/MAN-04199-001_002_02.pdf.

[64] Genius™ Digital Diagnostics System. https://www.hologic.com/hologic-products/cytology/genius-digital-diagnostics-system.

[65] CytoProcessor®. https://datexim.ai/cytoprocessor/.

[66] ScanAI Digital Pathology Platform. https://scanome.com/services/scanai-digital-pathology-platform/.

[67] EVApro. https://www.mobileodt.com/products/eva-pro/.

[68] Paige.ai. https://www.paige.ai/.

[69] CytoSiA—To Advance Cytology Screening Through Unmatched Image Quality and Artificial Intelligence Based Image Analysis Solution. https://www.optrascan.com/blogs/cytosia-to-advance-cytology-screening-through-unmatched-image-quality-and-artificial-intelligence-based-image-analysis-solution.

[70] BD FocalPoint™ GS Imaging System. https://www.bd.com/en-us/products-and-solutions/products/product-families/bd-focalpoint-gs-imaging-system.

[71] Lahrmann B., Keil A., Ruiz F.M., Clarke M.A., Egemen D., Grewal K.K., Grabe F.P., Bartels L., Krauthoff A., Ströbel P. (2025). Closing the Automation Gap: Robust AI for Dual-Stain Cervical Cancer Screening Triage. Res. Sq. [Preprint].

